# Opportunistic use of artificial intelligence with X-ray imaging for diagnosis of HIV status in tuberculosis patients in Uganda and Tanzania

**DOI:** 10.1371/journal.pdig.0000988

**Published:** 2025-09-02

**Authors:** Dmitrii Cherezov, Tanmoy Dam, Irene Najjingo, Margaret Mbabazi, Harriet Kisembo, Bruce Kirenga, Grace Soka, Esther Ngadaya, Sayoki Mfinanga, Anant Madabhushi

**Affiliations:** 1 Department of Biomedical Engineering, Georgia Institute of Technology and Emory University, Atlanta, Georgia, United States of America; 2 Makerere University, Kampala, Uganda; 3 Mulago National Referral and Teaching Hospital, Kampala, Uganda; 4 National Institute for Medical Research, Dar es Salaam, Tanzania; 5 Atlanta Veterans Administration Medical Center, Atlanta, Georgia, United States of America; Liverpool John Moores University - City Campus: Liverpool John Moores University, UNITED KINGDOM OF GREAT BRITAIN AND NORTHERN IRELAND

Human immunodeficiency virus (HIV) and tuberculosis (TB) remain prevalent, particularly in regions like Africa and India [1]. Many individuals with HIV are unaware of their status due to limited diagnostic resources [2,3]. Conventional HIV tests, such as laboratory-based antibody or antigen assays, are resource-intensive and often inaccessible in rural settings, forcing patients to travel extensively for diagnosis [4]. Thus, alternative diagnostic approaches are urgently needed.

Chest X-rays are routinely used for TB screening, a common co-infection in HIV-positive individuals. Given this frequent co-occurrence, chest X-rays present an opportunity for opportunistic HIV screening. Recent advancements in machine learning (ML) demonstrate significant potential for medical image analysis, including automated TB diagnosis from X-rays [5].

**Previous radiologic studies, including Frey and colleagues [6] and Haramati and colleagues [7], have shown that HIV co-infection in TB patients is associated with atypical chest X-ray patterns—such as reduced cavitation, lymphadenopathy, diffuse infiltrates, or miliary spread—especially in individuals with low CD4 counts. This biological and clinical evidence supports our model’s objective to detect radiographic features indicative of HIV-associated immunosuppression in TB-positive patients.**

This study evaluates whether artificial intelligence (AI) can opportunistically screen HIV status in TB patients via chest X-ray images [8]. We hypothesize that HIV induces subtle but detectable radiographic changes in TB patients, enabling ML detection of HIV status. **In sub-Saharan African healthcare settings, where access to laboratory testing is often limited, image-based assessments can assist in identifying TB patients who may show signs of HIV co-infection. Such tools may help prompt targeted HIV testing, enabling earlier diagnosis and more efficient use of clinical resources.**

A dataset of chest X-ray images was collected from 265 TB patients in Uganda, alongside clinical data including HIV status, age, sex, and smoking history. Among the patients, 74 were HIV-positive. The mean age was 40.45 years for HIV-positive patients and 43.28 years for HIV-negative patients. The dataset included 192 males (48 HIV-positive) and 73 females (26 HIV-positive). Smoking history was available for some patients: 10 current smokers (8 HIV-positive), 62 never smokers (28 HIV-positive), 36 former smokers (16 HIV-positive), and 157 with unknown smoking history (22 HIV-positive). **An additional dataset from Tanzania included chest X-ray images from 13 patients. Demographic data were not available for these patients. Among them, 6 were HIV-positive and 7 were HIV-negative.**

**Fig 1:**
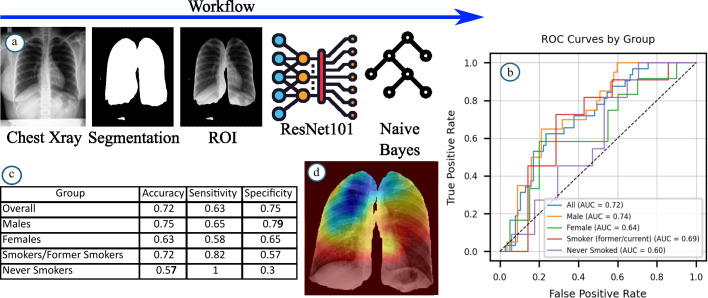
Workflow and performance details. **(a)** The workflow includes image processing steps such as lung segmentation and masking of the lung area on X-ray images, followed by extraction of deep features, and finally training a diagnostic model using a Random Forest classifier; **(b)** The AUROC performance metric was computed for the entire population as well as for different subpopulations: males, females, smokers, and never-smokers; **(c)** Accuracy, sensitivity, and specificity were computed for the selected populations. **(d)** Region within X-ray images whose texture correlates with HIV-positive status.

The data was split into training (60%) and testing (40%) sets, stratified by HIV status. Automated segmentation isolated lung regions from X-ray images. **The choice between deep learning and traditional ML methods was made in favor of the latter due to the limited dataset size. Future work should include the evaluation of diagnostic models trained using deep learning frameworks once a larger dataset becomes available.** A pre-trained ResNet-101 model extracted features from these segmented areas, capturing patterns potentially associated with HIV status. The Minimum Redundancy Maximum Relevance (mRMR) algorithm selected the most informative features. **To balance the training data, SMOTE was applied to generate 71 synthetic HIV-positive samples, resulting in a total of 242 instances (121 HIV-negative, 50 original HIV-positive instances, and 71 synthetic HIV-positive instances).** The workflow is shown in Fig 1a.

A Random Forest model, trained with features selected by mRMR, classified HIV status from X-ray features. This ensemble method constructs multiple decision trees, efficiently managing high-dimensional data. Hyperparameters were optimized via grid search and cross-validation. Model performance was evaluated using AUROC, sensitivity, and specificity.

The Random Forest model achieved an AUROC of 0.72, specificity of 0.75, and sensitivity of 0.63. Detailed results are illustrated in Fig 1b and 1c. **Because the selected features originate from high-dimensional latent representations within a deep network, their exact nature is abstract and not directly interpretable in clinical terms. To address this, we used GradCAM to provide visual explanations of the image regions most relevant to HIV-positive predictions. The saliency map shown in** Fig 1d **was generated using the GradCAM [9] algorithm for a randomly selected feature from the top-ranked training features, applied to the X-ray of a representative HIV-positive patient.**

Performance varied across subpopulations: AUROC was 0.74 for males versus 0.64 for females, and 0.69 for smokers compared to 0.60 for never smokers [10]. Higher performance in males may result from physiological differences or variations in X-ray quality [11]. Acknowledging these differences ensures equitable diagnostic utility across patient groups. Improved performance among smokers suggests smoking-related lung changes enhance model detection capability. Adjusting models for nonsmoking patients could further improve accuracy.

Overall, this study demonstrates AI’s potential for opportunistic HIV screening using chest X-rays obtained for TB diagnosis. Despite moderate accuracy, the findings support the feasibility of using ML to detect complex interactions between co-existing diseases like TB and HIV. Related work by Pyrros and colleagues [12] combined raw chest X-rays and electronic health records (EHR) to predict type 2 diabetes, whereas our approach relies solely on deep features from segmented lung images without EHR integration or end-to-end training.

Utilizing existing chest X-rays to identify high-risk patients could reduce reliance on costly laboratory-based diagnostics, generating substantial savings for healthcare systems in Africa. Early detection and treatment also curb disease progression and HIV transmission, reducing long-term healthcare costs associated with advanced HIV/AIDS management. **Observed differences in model performance across patient subgroups, such as higher diagnostic accuracy among smokers, may point toward specific target populations for which opportunistic diagnostic models are most applicable. Rather than assuming uniform application across all patients, future work should consider subgroup-specific deployment strategies to maximize clinical value.**

**The results of this study highlight the potential for opportunistic disease detection in diagnostic imaging, even when the primary clinical objective is unrelated. For instance, Frey and colleagues [6] demonstrated that deep learning can predict cardiovascular disease risk from low-dose lung cancer screening CTs, and Hiremath and colleagues [13] showed that lung shape features derived from chest CT scans correlate with COVID-19 severity. While promising, such applications require further investigation to assess potential biases, particularly when analyzing patients with known primary diagnoses, as in our case with TB.**

**We believe that dataset curation efforts—particularly in the context of public dataset releases—should aim to capture not only labels related to the primary disease but also comprehensive descriptors of the patient’s broader clinical status. Such enriched datasets would facilitate a wide range of diagnostic applications, including the detection of comorbid conditions and risk profiling.**

We acknowledge study limitations, including a relatively small sample size that may impact model robustness. Additionally, questions remain about the generalizability of AI-derived radiographic patterns due to the complex interaction between HIV and TB, which individually and jointly affect lung pathology. Further research is necessary to confirm whether these AI-derived patterns generalize across diverse populations. Nonetheless, our findings underscore AI’s potential to enhance healthcare delivery by providing rapid, noninvasive HIV diagnosis in TB patients in resource-constrained environments.

**Identification and comparison of radiographic features in HIV-positive and HIV-negative TB patients may hold substantial clinical relevance, especially in supporting differential diagnosis and treatment prioritization. This line of research may also enhance our understanding of HIV’s impact on TB presentation and contribute to the development of population-specific diagnostic strategies. Further work with larger datasets could enhance the ability to relate AI-extracted features to medically interpretable radiographic patterns and improve generalizability across diverse populations.**

**Our study is one of the first to investigate whether HIV-associated changes in the radiographic appearance of TB can be captured using ML methods. By applying feature-based analysis to real-world X-ray data from sub-Saharan Africa, we highlight the potential of opportunistic HIV risk detection during TB diagnosis in low-resource settings.**

**This study was supported by NIH/NCI U54CA254566, and only deidentified chest X-ray images were used for analysis. All image de-identification was performed using DICOM Cleaner software to ensure the removal of personal identifiers. As a result, the study was deemed exempt from IRB review and classified as non-human subjects research according to institutional guidelines.**
