# Correction: Opportunistic use of artificial intelligence with X-ray imaging for diagnosis of HIV status in tuberculosis patients in Uganda and Tanzania

**DOI:** 10.1371/journal.pdig.0001164

**Published:** 2025-12-23

**Authors:** The PLOS One Staff

## Notice of Republication

This article was republished on November 05, 2025, to correct an error in Figure 1C, which did not display correctly due to a production issue. The figure has been replaced with the correct version. The publisher apologizes for the error. Please download this article again to view the correct version. The originally published, uncorrected article and the republished, corrected articles are provided here for reference.

## Supporting information

S1 File Originally published, uncorrected article. (PDF)

S2 File Republished, corrected article. (PDF)
