# Supplementary material for: Correction: Opportunistic use of artificial intelligence with X-ray imaging for diagnosis of HIV status in tuberculosis patients in Uganda and Tanzania
Source: PLOS Digit Health. 2025 Dec 23;4(12):e0001164. doi: 10.1371/journal.pdig.0001164 (PMC12725681; doi:10.1371/journal.pdig.0001164)
Supplement: S2 File — (PDF) [file pdig.0001164.s002.pdf]

PLOS Digital Health

OPINION

Opportunistic use of artificial intelligence with X-ray imaging for diagnosis of HIV status in tuberculosis patients in Uganda and Tanzania

Dmitrii Cherezov[1], Tanmoy Dam[1], Irene Najjingo[2], Margaret Mbabazi[2], Harriet Kisembo[3], Bruce Kirenga[2], Grace Soka[4], Esther Ngadaya[4], Sayoki Mfinanga[4], Anant Madabhushi[1,5]*

1 Department of Biomedical Engineering, Georgia Institute of Technology and Emory University, Atlanta, Georgia, United States of America, **2** Makerere University, Kampala, Uganda, **3** Mulago National Referral and Teaching Hospital, Kampala, Uganda, **4** National Institute for Medical Research, Dar es Salaam, Tanzania, **5** Atlanta Veterans Administration Medical Center, Atlanta, Georgia, United States of America

* anant.madabhushi@emory.edu

Check for updates

OPEN ACCESS

Citation: Cherezov D, Dam T, Najjingo I, Mbabazi M, Kisembo H, Kirenga B, et al. (2025) Opportunistic use of artificial intelligence with X-ray imaging for diagnosis of HIV status in tuberculosis patients in Uganda and Tanzania. PLOS Digit Health 4(9): e0000988. https://doi.org/10.1371/journal.pdig.0000988

Editor: Dhiya Al-Jumeily OBE, Liverpool John Moores University - City Campus: Liverpool John Moores University, UNITED KINGDOM OF GREAT BRITAIN AND NORTHERN IRELAND

Published: September 2, 2025

Copyright: © 2025 Cherezov et al. This is an open access article distributed under the terms of the Creative Commons Attribution License, which permits unrestricted use, distribution, and reproduction in any medium, provided the original author and source are credited.

Funding: Research reported in this publication was supported by the National Cancer Institute under award numbers R01CA249992-01A1, R01CA216579-01A1, R01CA257612-01A1, R01CA264017-01, R01CA268287-01A1, U01CA113913-16A1 U01CA239055-01, U01CA269181-01, U24CA274494-01, and

Human immunodeficiency virus (HIV) and tuberculosis (TB) remain prevalent, particularly in regions like Africa and India [1]. Many individuals with HIV are unaware of their status due to limited diagnostic resources [2,3]. Conventional HIV tests, such as laboratory-based antibody or antigen assays, are resource-intensive and often inaccessible in rural settings, forcing patients to travel extensively for diagnosis [4]. Thus, alternative diagnostic approaches are urgently needed.

Chest X-rays are routinely used for TB screening, a common co-infection in HIV-positive individuals. Given this frequent co-occurrence, chest X-rays present an opportunity for opportunistic HIV screening. Recent advancements in machine learning (ML) demonstrate significant potential for medical image analysis, including automated TB diagnosis from X-rays [5].

Previous radiologic studies, including Frey and colleagues [6] and Haramati and colleagues [7], have shown that HIV co-infection in TB patients is associated with atypical chest X-ray patterns—such as reduced cavitation, lymphadenopathy, diffuse infiltrates, or miliary spread—especially in individuals with low CD4 counts. This biological and clinical evidence supports our model's objective to detect radiographic features indicative of HIV-associated immunosuppression in TB-positive patients.

This study evaluates whether artificial intelligence (AI) can opportunistically screen HIV status in TB patients via chest X-ray images [8]. We hypothesize that HIV induces subtle but detectable radiographic changes in TB patients, enabling ML detection of HIV status. **In sub-Saharan African healthcare settings, where access to laboratory testing is often limited, image-based assessments can assist in identifying TB patients who may show signs of HIV co-infection. Such tools may help prompt targeted HIV testing, enabling earlier diagnosis and more efficient use of clinical resources.**

A dataset of chest X-ray images was collected from 265 TB patients in Uganda, alongside clinical data including HIV status, age, sex, and smoking history.

U54CA254566-01 (to AM); the National Heart, Lung and Blood Institute under award numbers R01HL151277-01A1 and R01HL158071-01A1 (to AM); the National Institute of Allergy and Infectuous Diseases (R01AI175555) (to AM); the National Institute of Dental and Crainofacial Research (R21DE032344-01) (to AM); the National Library of Medicine (R01LM013864-01A1) (to AM); the National Institute on Aging (R01AG089759) (to AM); the National Institute of Diabetes and Digestive and Kidney Diseases (R01DK118431) (to AM); the Kidney Mapping and Atlas Project (KMAP) under U01DK133090-01 (to AM); the United States Department of Veterans Affairs VA Merit Review award (IBX004121) (to AM); the VA Biomedical Laboratory Research and Development Service under awards I01CX002622, I01CX002776, and IK6BX006185 (to AM); the VA Research and Development Office through the Lung Precicision Oncology Program (LPOP-L0021) (to AM); the Office of the Assistant Secretary of Defense for Health Affairs through the Prostate Cancer Research Program (W81XWH-15–1-0558, W81XWH-20–1-0851, W81XWH-21-1-0160) (to AM); and sponsored research agreements from Astrazeneca, Bristol Myers Squibb, the Prevent Cancer Foundation, Innovation in Cancer Informatics, and the Scott Mackenzie Foundation. The content is solely the responsibility of the authors and does not necessarily represent the official views of the National Institutes of Health, the U.S. Department of Veterans Affairs, the Department of Defense, or the United States Government. The funders had no role in study design, data collection and analysis, decision to publish, or preparation of the manuscript.

Competing interests: I have read the journal's policy and the authors of this manuscript have the following competing interests: Dr. Madabhushi is an equity holder in Picture Health, Elucid Bioimaging, and Inspirata Inc. Currently, he serves on the advisory board of Picture Health, Aiforia Inc., and SimBioSys. He also currently consults for SimBioSys. He also has sponsored research agreements with AstraZeneca, Boehringer-Ingelheim, Eli-Lilly, and Bristol Myers-Squibb. His technology has been licensed to Picture Health and Elucid Bioimaging. He is also involved in 3 different R01 grants with Inspirata Inc. He also serves as a member for the Frederick National Laboratory Advisory Committee. Other authors declare that they have no financial interests.

Among the patients, 74 were HIV-positive. The mean age was 40.45 years for HIV-positive patients and 43.28 years for HIV-negative patients. The dataset included 192 males (48 HIV-positive) and 73 females (26 HIV-positive). Smoking history was available for some patients: 10 current smokers (8 HIV-positive), 62 never smokers (28 HIV-positive), 36 former smokers (16 HIV-positive), and 157 with unknown smoking history (22 HIV-positive). **An additional dataset from Tanzania included chest X-ray images from 13 patients. Demographic data were not available for these patients. Among them, 6 were HIV-positive and 7 were HIV-negative.**

The data was split into training (60%) and testing (40%) sets, stratified by HIV status. Automated segmentation isolated lung regions from X-ray images. **The choice between deep learning and traditional ML methods was made in favor of the latter due to the limited dataset size. Future work should include the evaluation of diagnostic models trained using deep learning frameworks once a larger dataset becomes available.** A pre-trained ResNet-101 model extracted features from these segmented areas, capturing patterns potentially associated with HIV status. The Minimum Redundancy Maximum Relevance (mRMR) algorithm selected the most informative features. **To balance the training data, SMOTE was applied to generate 71 synthetic HIV-positive samples, resulting in a total of 242 instances (121 HIV-negative, 50 original HIV-positive instances, and 71 synthetic HIV-positive instances).** The workflow is shown in Fig 1a.

A Random Forest model, trained with features selected by mRMR, classified HIV status from X-ray features. This ensemble method constructs multiple decision trees, efficiently managing high-dimensional data. Hyperparameters were optimized via grid search and cross-validation. Model performance was evaluated using AUROC, sensitivity, and specificity.

The Random Forest model achieved an AUROC of 0.72, specificity of 0.75, and sensitivity of 0.63. Detailed results are illustrated in Fig 1b and 1c. **Because the selected features originate from high-dimensional latent representations within a deep network, their exact nature is abstract and not directly interpretable in clinical terms. To address this, we used GradCAM to provide visual explanations of the image regions most relevant to HIV-positive predictions. The saliency map shown in Fig 1d was generated using the GradCAM [9] algorithm for a randomly selected feature from the top-ranked training features, applied to the X-ray of a representative HIV-positive patient.**

Performance varied across subpopulations: AUROC was 0.74 for males versus 0.64 for females, and 0.69 for smokers compared to 0.60 for never smokers [10]. Higher performance in males may result from physiological differences or variations in X-ray quality [11]. Acknowledging these differences ensures equitable diagnostic utility across patient groups. Improved performance among smokers suggests smoking-related lung changes enhance model detection capability. Adjusting models for nonsmoking patients could further improve accuracy.

Overall, this study demonstrates AI's potential for opportunistic HIV screening using chest X-rays obtained for TB diagnosis. Despite moderate accuracy, the

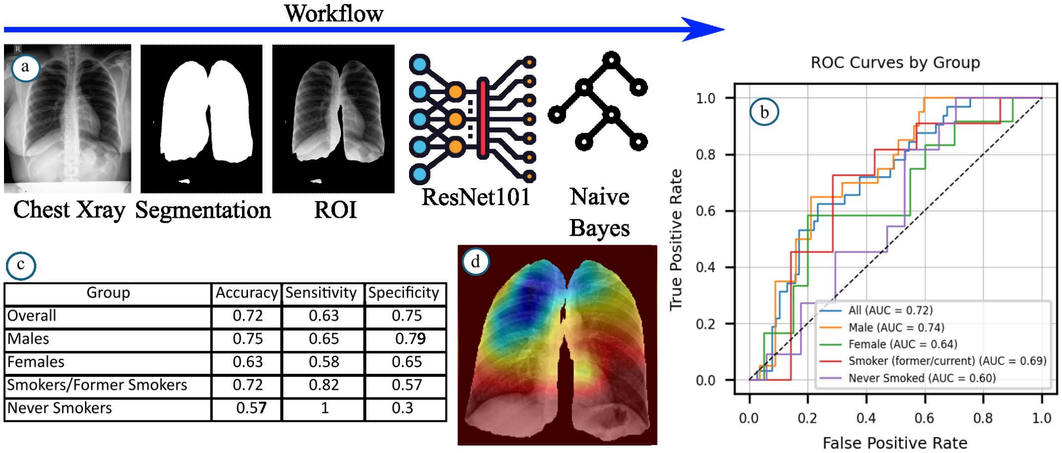

Group	Accuracy	Sensitivity	Specificity
Overall	0.72	0.63	0.75
Males	0.75	0.65	0.79
Females	0.63	0.58	0.65
Smokers/Former Smokers	0.72	0.82	0.57
Never Smokers	0.57	1	0.3

Fig 1. Workflow and performance details. **(a)** The workflow includes image processing steps such as lung segmentation and masking of the lung area on X-ray images, followed by extraction of deep features, and finally training a diagnostic model using a Random Forest classifier; **(b)** The AUROC performance metric was computed for the entire population as well as for different subpopulations: males, females, smokers, and never-smokers; **(c)** Accuracy, sensitivity, and specificity were computed for the selected populations. **(d)** Region within X-ray images whose texture correlates with HIV-positive status.

https://doi.org/10.1371/journal.pdig.0000988.g001

findings support the feasibility of using ML to detect complex interactions between co-existing diseases like TB and HIV. Related work by Pyrros and colleagues [12] combined raw chest X-rays and electronic health records (EHR) to predict type 2 diabetes, whereas our approach relies solely on deep features from segmented lung images without EHR integration or end-to-end training.

Utilizing existing chest X-rays to identify high-risk patients could reduce reliance on costly laboratory-based diagnostics, generating substantial savings for healthcare systems in Africa. Early detection and treatment also curb disease progression and HIV transmission, reducing long-term healthcare costs associated with advanced HIV/AIDS management. **Observed differences in model performance across patient subgroups, such as higher diagnostic accuracy among smokers, may point toward specific target populations for which opportunistic diagnostic models are most applicable. Rather than assuming uniform application across all patients, future work should consider subgroup-specific deployment strategies to maximize clinical value.**

The results of this study highlight the potential for opportunistic disease detection in diagnostic imaging, even when the primary clinical objective is unrelated. For instance, Frey and colleagues [6] demonstrated that deep learning can predict cardiovascular disease risk from low-dose lung cancer screening CTs, and Hiremath and colleagues [13] showed that lung shape features derived from chest CT scans correlate with COVID-19 severity. While promising, such applications require further investigation to assess potential biases, particularly when analyzing patients with known primary diagnoses, as in our case with TB.

We believe that dataset curation efforts—particularly in the context of public dataset releases—should aim to capture not only labels related to the primary disease but also comprehensive descriptors of the patient's broader clinical status. Such enriched datasets would facilitate a wide range of diagnostic applications, including the detection of comorbid conditions and risk profiling.

We acknowledge study limitations, including a relatively small sample size that may impact model robustness. Additionally, questions remain about the generalizability of AI-derived radiographic patterns due to the complex interaction between HIV and TB, which individually and jointly affect lung pathology. Further research is necessary to confirm whether these AI-derived patterns generalize across diverse populations. Nonetheless, our findings underscore AI's potential

to enhance healthcare delivery by providing rapid, noninvasive HIV diagnosis in TB patients in resource-constrained environments.

Identification and comparison of radiographic features in HIV-positive and HIV-negative TB patients may hold substantial clinical relevance, especially in supporting differential diagnosis and treatment prioritization. This line of research may also enhance our understanding of HIV's impact on TB presentation and contribute to the development of population-specific diagnostic strategies. Further work with larger datasets could enhance the ability to relate AI-extracted features to medically interpretable radiographic patterns and improve generalizability across diverse populations.

Our study is one of the first to investigate whether HIV-associated changes in the radiographic appearance of TB can be captured using ML methods. By applying feature-based analysis to real-world X-ray data from sub-Saharan Africa, we highlight the potential of opportunistic HIV risk detection during TB diagnosis in low-resource settings.

This study was supported by NIH/NCI U54CA254566, and only deidentified chest X-ray images were used for analysis. All image de-identification was performed using DICOM Cleaner software to ensure the removal of personal identifiers. As a result, the study was deemed exempt from IRB review and classified as non-human subjects research according to institutional guidelines.

Author contributions

Conceptualization: Dmitrii Cherezov, Anant Madabhushi.

Data curation: Irene Najjingo, Margaret Mbabazi, Harriet Kisembo, Bruce Kirenga.

Formal analysis: Dmitrii Cherezov, Tanmoy Dam, Anant Madabhushi.

Funding acquisition: Anant Madabhushi.

Investigation: Dmitrii Cherezov.

Methodology: Dmitrii Cherezov.

Project administration: Anant Madabhushi.

Resources: Anant Madabhushi.

Software: Dmitrii Cherezov, Tanmoy Dam.

Supervision: Anant Madabhushi.

Validation: Anant Madabhushi.

Visualization: Dmitrii Cherezov.

Writing – original draft: Dmitrii Cherezov.

Writing – review & editing: Anant Madabhushi.

References

1. Bagcchi S. WHO's global tuberculosis report 2022. Lancet Microbe. 2023;4(1):e20.
2. Fan S, Abulizi A, You Y, Huang C, Yimit Y, Li Q, et al. Predicting hospitalization costs for pulmonary tuberculosis patients based on machine learning. BMC Infect Dis. 2024;24(1):875. https://doi.org/10.1186/s12879-024-09771-6 PMID: 39198742
3. Veesa KS, John KR, Moonan PK, Kaliappan SP, Manjunath K, Sagili KD, et al. Diagnostic pathways and direct medical costs incurred by new adult pulmonary tuberculosis patients prior to anti-tuberculosis treatment—Tamil Nadu, India. PLoS One. 2018;13(2):e0191591. https://doi.org/10.1371/journal.pone.0191591 PMID: 29414980
4. Togawa K, Anderson BO, Foerster M, Galukande M, Zietsman A, Pontac J, et al. Geospatial barriers to healthcare access for breast cancer diagnosis in sub-Saharan African settings: the African breast cancer-disparities in outcomes cohort study. Int J Cancer. 2021;148(9):2212–26. https://doi.org/10.1002/ijc.33400 PMID: 33197280

5. Galić I, Habijan M, Leventić H, Romić K. Machine learning empowering personalized medicine: a comprehensive review of medical image analysis methods. Electronics. 2023;12(21):4411.
6. Frey V, Phi Van VD, Fehr JS, Ledergerber B, Sekaggya-Wiltshire C, Castelnuovo B, et al. Prospective evaluation of radiographic manifestations of tuberculosis in relationship with CD4 count in patients with HIV/AIDS. Medicine (Baltimore). 2023;102(7):e32917. https://doi.org/10.1097/MD.0000000000032917 PMID: 36800631
7. Haramati LB, Jenny-Avital ER, Alterman DD. Effect of HIV status on chest radiographic and CT findings in patients with tuberculosis. Clin Radiol. 1997;52(1):31–5. https://doi.org/10.1016/s0009-9260(97)80302-9 PMID: 9022577
8. Pawlowski A, Jansson M, Sköld M, Rottenberg ME, Källenius G. Tuberculosis and HIV co-infection. PLoS Pathog. 2012;8(2):e1002464. https://doi.org/10.1371/journal.ppat.1002464 PMID: 22363214
9. Selvaraju RR, Cogswell M, Das A, Vedantam R, Parikh D, Batra D. Grad-cam: Visual explanations from deep networks via gradient-based localization. In: Proceedings of the IEEE International conference on computer vision. 2017.
10. Konstantinidis I, Crothers K, Kunisaki KM, Drummond MB, Benfield T, Zar HJ, et al. HIV-associated lung disease. Nat Rev Dis Primers. 2023;9(1):39. https://doi.org/10.1038/s41572-023-00450-5 PMID: 37500684
11. Ezeamama AE, Mupere E, Oloya J, Martinez L, Kakaire R, Yin X, et al. Age, sex, and nutritional status modify the CD4+ T-cell recovery rate in HIV-tuberculosis co-infected patients on combination antiretroviral therapy. Int J Infect Dis. 2015;35:73–9. https://doi.org/10.1016/j.ijid.2015.04.008 PMID: 25910854
12. Pyrros A, Borstelmann SM, Mantravadi R, Zaiman Z, Thomas K, Price B, et al. Opportunistic detection of type 2 diabetes using deep learning from frontal chest radiographs. Nat Commun. 2023;14(1):4039. https://doi.org/10.1038/s41467-023-39631-x PMID: 37419921
13. Hiremath A, Viswanathan VS, Bera K, Shiradkar R, Yuan L, Armitage K, et al. Deep learning reveals lung shape differences on baseline chest CT between mild and severe COVID-19: a multi-site retrospective study. Comput Biol Med. 2024;177:108643. https://doi.org/10.1016/j.compbiomed.2024.108643 PMID: 38815485
